# Exploring the Lipid World Hypothesis: A Novel Scenario of Self-Sustained Darwinian Evolution of the Liposomes

**DOI:** 10.1089/ast.2021.0161

**Published:** 2023-03-01

**Authors:** Vladimir Subbotin, Gennady Fiksel

**Affiliations:** ^1^ Department of Human Oncology, University of Wisconsin-Madison, Madison, Wisconsin, USA.; ^2^ Department of Nuclear Engineering and Radiological Sciences, University of Michigan, Ann Arbor, Michigan, USA.

**Keywords:** Origin of life, Lipid World, Heavy liposomes, Darwinian evolution of liposomes, Solar UV radiation, Day/night cycle, Gravity

## Abstract

According to the Lipid World hypothesis, life on Earth originated with the emergence of amphiphilic assemblies in the form of lipid micelles and vesicles (liposomes). However, the mechanism of appearance of the information molecules (ribozymes/RNA) accompanying that process, considered obligatory for Darwinian evolution, is unclear. We propose a novel scenario of self-sustained Darwinian evolution of the liposomes driven by ever-present natural phenomena: solar UV radiation, day/night cycle, gravity, and the formation of liposomes in an aqueous media. The central tenet of this scenario is the liposomes' encapsulation of the heavy solutes, followed by their gravitational submerging in the water. The submerged liposomes, being protected from the damaging UV radiation, acquire the longevity necessary for autocatalytic replication of amphiphiles, their mutation, and the selection of those amphiphilic assemblies that provide the greatest membrane stability. These two sets of adaptive compositional information (heavy content and amphiphilic assemblies design) generate a population of liposomes with self-replication/reproduction properties, which are amendable to mutation, inheritance, and selection, thereby establishing Darwinian progression. Temporary and spatial expansion of this liposomal population will provide the basis for the next evolutionary step—a transition of accidentally entrapped RNA precursor molecules into complex functional molecules, such as ribozymes/RNA.

The gap between complex self-organizing phenomena (physico-chemical dissipative structures) and the simplest biological entities we know of today is too big to be bridged without some intermediate stage(s). — Ruiz-Mirazo *et al.* (1999)

The first living systems to persist on Earth are shrouded in mystery, and the first to persist may not have been the first to exist. — Schulze-Makuch and Irwin (2018)

The recognized models of life's origin on Earth originate from the Oparin-Haldane hypothesis (Опapин, 1924; Haldane, 1929) and assume the occurrence of several events that must align strictly consecutively in time and space, that is, appear as a multistage process. One example is the RNA World hypothesis, that is, the synthesis of purines and pyrimidine sugars, followed by the synthesis of nucleosides with subsequent polymerization, recombination, folding, and finally, the emergence of the first RNA replicase.

However, Oparin (1974) reasonably argued that “the probability that system A will necessarily change to produce system B is very low. But since the probabilities are to be multiplied rather than summed, any multistage process (even the appearance of complex organic substrates) seems to be extremely improbable.” Orgel (1998) also expressed a strong skepticism regarding spontaneous co-occurrence of multistage processes: “In my opinion, there is no basis in known chemistry for the belief that long sequences of reactions can organize spontaneously—and every reason to believe that they cannot.” These and other limitations (Kauffman, 2013; Walker and Davies, 2013) of the RNA-first hypothesis are well known and are described by both supportive and critical analyses (*e.g.,* Benner *et al.,* 2012; Bernhardt, 2012; Robertson and Joyce, 2012; Benner, 2014; Guttenberg *et al.,* 2017).

However, the major obstacle to any non-lipid-first hypothesis (*i.e.,* non-Lipid World hypotheses) of life's origin appears to be an unavoidable dilution and dissipation of the prebiotic molecules in an aqueous environment. While Patel *et al*. (2015) suggested that “all the cellular subsystems could have arisen simultaneously through common chemistry,” there are no forces/affinities that could keep together the RNA precursors, peptides, and lipids. Therefore, the Brownian motion and the aqueous movement will ultimately lead to their diversion and dilution. Besides, that hypothesis (Patel *et al.,* 2015) does not take into account the high likelihood of the same molecules being involved in alternative reactions (Schulze-Makuch and Irwin, 2018).

The puzzle of temporal and spatial coincidence of the prebiotic and biotic molecules was addressed by suggesting that surfaces of certain minerals might attract and orient the prebiotic and biotic molecules and even initiate effective sequential catalysis, thereby supporting highly regiospecific reactions (Cairns-Smith, 1990; Ferris and Ertem, 1993; Ferris *et al.,* 1996; Robertson and Joyce, 2012).

However, even considering the highly regiospecific reactions on minerals' surfaces (Ferris and Ertem, 1993; Ferris *et al.,* 1996; Robertson and Joyce, 2012), the essential biotic molecules have to be compartmentalized or encapsulated because only after the encapsulation do the above components have a chance to exist and function as a unit that could be further subjected to inheritable modifications, replication, and selection, that is, to be a subject of Darwinian evolution from which life emerged (Szostak *et al.,* 2001; Kunnev, 2020). The founders of the heterotrophic theory recognized an obligatory necessity of the amalgamation of prebiotic molecules in space and time for life's origin. In a description of the primordial soup, Oparin writes, “Thus, in this initially arising solution of complex and diverse organic substances, there was still no organization, both in space and in time” (Опapин, 1941, p 129, our translation). To circumvent this lack of space and time unity, Oparin hypothesized the formation of colloid particles or coacervates, which can compartmentalize ingredients of the primordial soup (Oparin, 1941, 1953). At the same time, Haldane suggested, “The critical event which may best be called the origin of life was the enclosure of several self-reproducing polymers within a semipermeable membrane” (Haldane, 1954). The mandatory necessity of the compartmentalizing systems was emphasized in 1971 by Eigen (Eigen, 1971) and Tibor Gánti (Gánti, 1971—English translation: Gánti, 2003). Recent analysis on the origin of life by Stuart Kauffman also stresses that any progression of information molecules toward life is plausible only “if housed in dividing compartments such as dividing liposomes” (Kauffman, 2011).

Since the presence of the encapsulating structures appears as the theoretical must (*e.g.,* Szostak *et al.,* 2001; Kauffman, 2011), in all analyses on the origin of life, the compartmentalizing structure is depicted in the form of a lipid bilayer vesicle or liposome. Particularly, in RNA World models, the essential compartmentalizing structure is suggested as spontaneously and inadvertently co-occurring with the ribozymes/RNA event, as depicted in numerous publications (*e.g.,* Pressman *et al.,* 2015; Le Vay and Mutschler, 2019; Figs. 1 and 2 in this article).

**FIG. 1.**
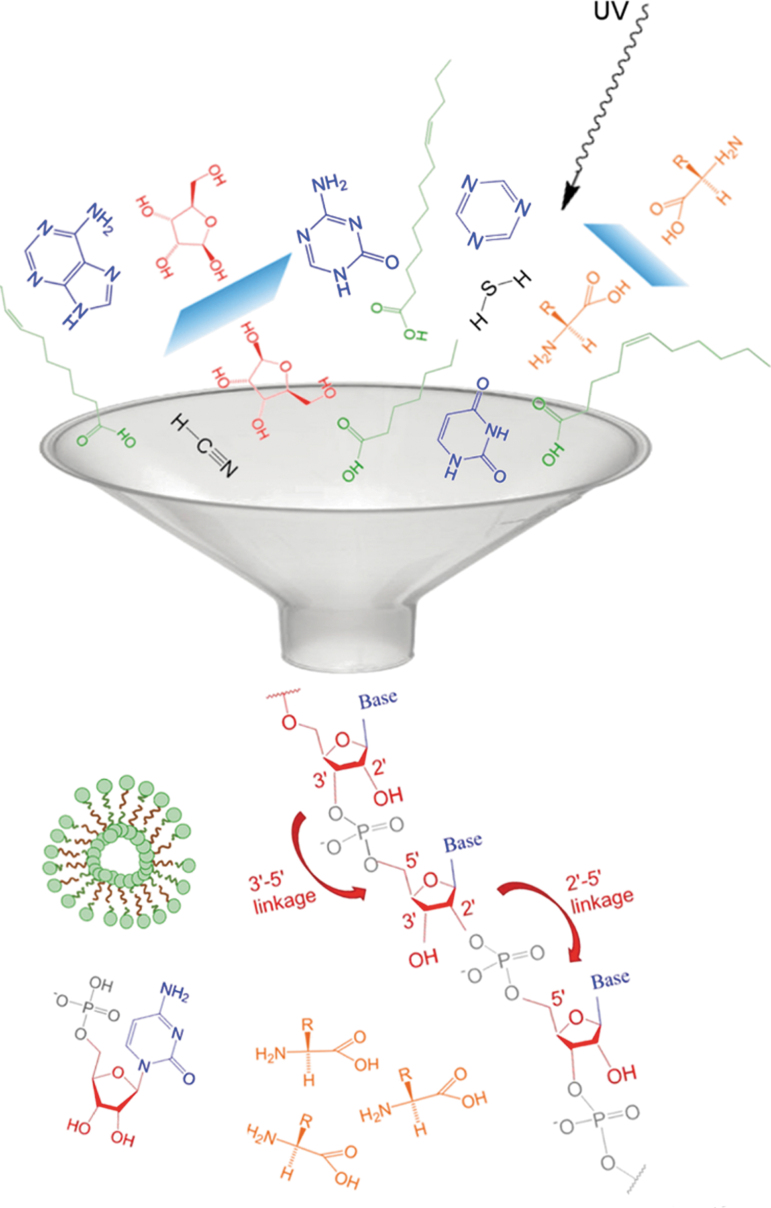
Inadvertent occurrence of a liposome. Reproduced from Pressman *et al.* (2015), with permission.

**FIG. 2.**
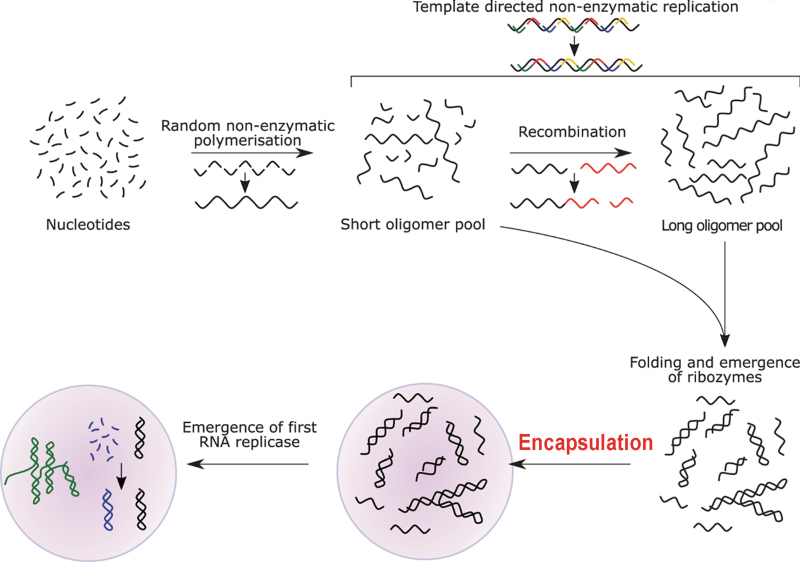
Inadvertent occurrence of the compartmentalizing structure (liposome). Modified (bold red font) and reproduced from Le Vay and Mutschler (2019), under Creative Commons Attribution License 4.0 (CC BY).

The seeming convenience of such modeling contradicts common sense because simultaneous co-emergence in time and space of two probable events, the ribozymes/RNA event and the liposome formation, appears implausible (see above).

Therefore, we share the opinion that in the RNA First scenario, the emerged ribozymes and/or RNA are destined to fail the further advancement due to inevitable dilution and dissipation of the prebiotic and biotic molecules in a free aqueous environment. The simultaneous co-emergence of a liposome appears improbable even when considering the self-sustained RNA enzyme replication (Lincoln and Joyce, 2009). Common sense dictates that one of the counterparts must emerge first, survive, and propagate for a long time in abundance. Ribozymes and/or RNA cannot survive and evolve by themselves without a liposome. A logical question is, can liposomes emerge, survive, and then evolve on their own?

Many notable analyses were published in favor of such a scenario, also known as the Lipid World, starting with a pioneering publication by Morowitz *et al.* (1988) suggesting that amphiphilic membrane vesicles produce self-similar assemblies by breaking into smaller vesicles, followed by descendant vesicles' growth. This hypothesis was further elaborated in theoretical and experimental studies by several research groups (*e.g.,* Luisi *et al.,* 1999; Segré and Lancet, 2000; Segré *et al.,* 2001; Szostak *et al.,* 2001; Stano and Luisi, 2010; Damer and Deamer, 2015; Deamer, 2017, 2018).

The Lipid World research emphasizes the idea that a design of amphiphilic assemblies can be a source of inheritable information by itself. In one of the pioneering articles on the Lipid World hypothesis, Segré *et al.* (2001) proposed that “crucial steps in the origin of life might have been carried out by lipid-like molecules alone, potentially prior to the emergence of polynucleic acids and polypeptides.” Furthermore, the authors suggested that “heterogeneous autocatalytic lipozymes with defined internal compositions might have been gradually selected out of an initial highly complex repertoire of micelles and vesicles formed spontaneously by abiotic processes. That would entail information content as well as a capacity to undergo natural selection, as delineated below….” The authors have emphasized that this phenomenon belongs “to the class of phenotypical replicators … rather than digital inheritance.”

However, this model has a factual impediment: solar UV radiation. Phospholipid layers and vesicles are always formed at the air-water interface, and they would be inevitably destroyed during daytime by solar UV radiation, which at the time of primitive Earth was of a shorter wavelength (Miller and Urey, 1959) and had an intensity 3–4 orders of magnitude higher than at present (Zahnle and Walker, 1982; Cockell, 1998, 2000).

Here we would like to deliberate on this problem and offer a new model, a new hypothesis within the Lipid World framework that circumvents that obstacle. A simple quantitative theoretical basis discussed below suggests possible experiments to evaluate the hypothesis.

## Our Model in Brief

Amphiphilic macromolecules, for example, phospholipids, being released in an aqueous environment inevitably ascend and concentrate at the water-air interface. After reaching a critical concentration, the phospholipids undergo self-assembly into the Langmuir layer and bilayer, micelles, and liposomes at the water-air interface, where these assemblies are affected by solar UV radiation. The UV radiation decomposes all the assemblies into separate phospholipid molecules, forming a new Langmuir layer. This cycle of self-assembly/destruction is repeated every day when the Sun comes up, with a half-life of any assembly of about 12 h (nighttime). Provided that during the nighttime liposomogenesis the liposomes encapsulate a solute heavier than the surrounding media, for example, a solute of amino acids or sugars, those heavy liposomes will descend from a water-air interface to the bottom of the pool and become protected from UV by water. Every nighttime will add new heavy liposomes to the liposomal congregation at the bottom of the pool. The submerged liposomes, being protected from the damaging UV radiation, acquire the longevity necessary for autocatalytic replication of amphiphiles and, through their mutation and the selection of those amphiphilic assemblies, achieve the highest membrane stability.

## The Extended Version of Our Model

We want to start with a quotation from Martin Gerard Rutten's work (Rutten, 1957), whose notion is extremely important for our hypothesis. Rutten writes:

Geologic science is mainly built upon the Principle of Actualism. That implies that the same natural processes, found now in operation in the atmosphere, hydrosphere, and lithosphere, have been in operation throughout most of geologic history. Intensities varied, both in time and geographically, but no other, mysterious processes can be postulated in Earth's history as long as one bases this history on an actualistic conception.

We add that regarding the origin of life on Earth, the following natural processes should also be considered: solar UV radiation, day/night cycle, gravity, and the natural formation of amphiphiles and bilayer vesicles in aqueous media. While the presence of the first three processes is evident, the fourth process, particularly the formation of phospholipid bilayer vesicles, requires substantiation.

The classical view on phospholipid membrane assembly suggests that phospholipid synthesis is catalyzed by membrane-bound enzymes, which themselves require preexisting membranes (Szathmáry, 1999; Bhattacharya *et al.,* 2019) and enzymes (Exterkate *et al.,* 2018) for function, which put the problem into the “chicken or egg” category. However, enzyme-free natural reactions leading to fatty acids synthesis and self-assembled vesicles are described for various experimental conditions (*e.g.,* Bonhorst *et al.,* 1948; Gebicki and Hicks, 1973; Simoneit, 1995; Apel *et al.,* 2002), including the abiotic synthesis of protocell-like vesicles (Budin *et al.,* 2009). The abiotic synthesis of hydrocarbons was suggested under natural conditions of hydrothermal fields (Proskurowski *et al.,* 2008) and was shown in experiments with hydrothermal reactions of pyruvic acid (Hazen and Deamer, 2007). Recent observations have shown that experimental conditions similar to natural, for example, at soda lakes and hydrothermal oceanic vents, facilitate the enzyme-free synthesis of phospholipids in water (Liu *et al.,* 2020). (Note: the extraterrestrial origin of amphiphilic molecules is out of the scope of the hypothesis, though such a source is obvious [*e.g.,* Deamer and Pashley, 1989].)

Based on this discovery (Liu *et al.,* 2020), we can further suggest that when phospholipid molecules are continuously released into water, they inevitably form a thin film at the air-water interface, the so-called Langmuir monolayer (Kaganer *et al.,* 1999; Giner-Casares *et al.,* 2014). Once the surface of the water becomes saturated with phospholipids, a spontaneous formation of a floating phospholipid bilayer and bilayer vesicles, or liposomes, encapsulating the surrounding media takes place (Ivkov, 1986; Marsh, 2012; Lombardo *et al.,* 2015; Su *et al.,* 2020). The formation of liposomes, or liposomogenesis, is an entirely physical process (Walde, 2006) and occurs spontaneously (Edwards *et al.,* 1996; Lasic *et al.,* 2001; Antonietti and Förster, 2003; Phapal *et al.,* 2017) or under experimental conditions (Bangham *et al.,* 1965); the liposomogenesis may include a micellar stage (Kahana and Lancet, 2021).

The hydrocarbon chains in lipid bilayers are packed to a density equal to or less than that of pure water (Sheetz and Chan, 1972; Nagle and Wilkinson, 1978; Dill and Flory, 1981). Therefore, the liposomes remain at the air-water interface (Andre *et al.,* 1991; Ivanova *et al.,* 1991; Launois-Surpas *et al.,* 1992; Kulkarni and Brown, 1994; Raneva *et al.,* 1995; Heurtault *et al.,* 2003; Pichot *et al.,* 2013; Staton and Dungan, 2018; Bai *et al.,* 2019) where they can be affected by the solar UV radiation.

Solar UV radiation is universally considered an important force for life's origin on Earth. However, multiple views on the consequences of this force vary wildly. For example, Oparin considered the effect of UV to be destructive for life matter (Oparin, 1941), and this view is still upheld (Hazen, 2005; Messerotti and Chela-Flores, 2006; Todd *et al.,* 2021). On the other hand, Haldane (1929) writes, “when ultraviolet light acts on a mixture of water, carbon dioxide, and ammonia, a wide variety of organic substances are made, including sugars and apparently some of the materials from which proteins are built up.” Harold C. Urey (1952), deliberating the Oparin-Haldane hypothesis, also concludes that “the effects of electrical discharges on chemical substances leave no doubt that many compounds would be formed due to the absorption of ultraviolet.” A creative synthetic nature of UV for the transition from abiotic matter to biotic was one of the ideas behind Stanley Miller's famous experiment: “Electrical discharge was used to form free radicals instead of ultraviolet light because quartz absorbs wavelengths short enough to cause photo-dissociation of the gases” (Miller, 1953).

Nevertheless, the opinions regarding the UV effect on phospholipid bilayers and vesicles are univocal. Starting with the first studies on the electric breakdown of phospholipid membranes by UV (Potapenko *et al.,* 1972; Putvinskij *et al.,* 1977; Putvinsky *et al.,* 1979; Vladimirov *et al.,* 1986), it was confirmed in numerous experiments that UV decomposes liposomal phospholipid bilayers into separate phospholipid molecules (*e.g.,* Mandal and Chatterjee, 1980; Murphy, 1983; Agarwal *et al.,* 1987; Bose *et al.,* 1989; Pelle *et al.,* 1990; De *et al.,* 1993; Budai *et al.,* 2008; Mazari *et al.,* 2009; Pashkovskaya *et al.,* 2010; Pires *et al.,* 2019). It should be noted that in one experimental study a UV-induced formation of bilayer vesicles was observed (Veronese *et al.,* 1998), although the UV source used was lacking the shortwave spectrum (<300 nm) typical for primitive Earth (Miller and Urey, 1959).

Extrapolations from the historical evolution of the solar UV luminosity indicate that at the time of primitive Earth, the UV radiation was of a shorter wavelength (Miller and Urey, 1959) and had an intensity of 3–4 orders of magnitude higher than at present (Zahnle and Walker, 1982; Cockell, 1998, 2000). Oparin (1974) wrote:

The action of shortwave UV light or some other energy source upon the dissolved organic substances in the primitive ocean created a thermodynamic equilibrium. Numerous calculations have shown that under these conditions, nothing like the concentrated ‘primordial soup’ could appear, since under the action of UV the decomposition processes had to proceed much faster than the synthesis.

Therefore, the destructive effects of UV on prebiotic and biotic compounds have always been considered the major limiting factor for life originated on the water surface of early Earth (Sagan, 1973; Oparin, 1974; Cleaves and Miller, 1998; Messerotti and Chela-Flores, 2006; Björn *et al.,* 2015; Todd *et al.,* 2021). How can phospholipid vesicles, which are formed and tend to concentrate at the air-water interface, be spared from being dismantled into individual phospholipid molecules by strong UV radiation? Can we come up with a model of phospholipid vesicle protection from UV?

Martin Rutten gave a simple solution in his book *The Origin of Life by Natural Causes:*

One of the many paradoxes encountered in the early history of life lies in the fact that the same rays of the Sun which formed the building blocks of the molecules of life were lethal for life. Early life had, therefore, only limited environmental possibilities. It could be survived only when shielded by a thick layer of water…. (Rutten, 1971)

But how can the floating liposomes submerge into deeper layers of water?

It is well known that the liposomal entrapment of either lighter or heavier solutions than the aqueous media in which the liposomes are suspended results in the formation of either floating or submerging liposomes. Irrespective of the composition of the entrapped solution, the heavy liposomes would sink away from the air-water interface to deeper layers of water (Chakrabarti *et al.,* 1990, 1992; Veiro and Cullis, 1990; Kernen *et al.,* 1992; Shin *et al.,* 2005). As a matter of fact, loading liposomes with an aqueous media of variable specific gravity is a common approach in liposome separation techniques (*e.g.,* Chakrabarti *et al.,* 1990; Li *et al.,* 2006; Schulz *et al.,* 2009; Bian and De Camilli, 2019).

From the revolutionary Miller-Urey experiments (Miller, 1953; Miller and Urey, 1959) followed by many others (for review, see Lazcano and Bada, 2003), we know that, in addition to amino acids, numerous molecules heavier than water, for example simple sugars (Bada, 2004) and even simple peptides (Parker *et al.,* 2014), could be continuously synthesized in the prebiotic Earth atmosphere. These Miller-Urey molecules (MUm) may come into contact with the Langmuir layer and be encapsulated during liposomogenesis at the water-air interface during nighttime. The presence of MUm in the liposomal interior may change the liposomal buoyancy from a positive to a negative. The liposomes then may descend from the water-air interface. Additionally, the already formed liposomes can extract MUm from the surrounding media during the nighttime; such extraction was observed in experiments (de Souza *et al.,* 2014), even against the concentration gradient of these molecules (Sugiyama *et al.,* 2020), and this phenomenon is thought to be relevant to the origin of life (de Souza *et al.,* 2014).

Another source of heavy elements for the heavy liposomogenesis is plausible. Solutes of hydrogen sulfide, ferric iron, along with a variety of molecules, for example B, Zn, Mn, P, S, are assumed to be naturally present in primordial water (Ranjan *et al.,* 2018; Damer and Deamer, 2020; Van Kranendonk *et al.,* 2021). These Natural Primordial Heavy molecules (NPHm) could be carried by the upward water motion to the surface. That process can coincide with the liposomogenesis during nighttime, or NPHm can be extracted and concentrated by already formed liposomes by the mechanisms mentioned above (de Souza *et al.,* 2014; Sugiyama *et al.,* 2020).

While the liposomal entrapment of heavier molecules, coming from either the atmosphere or the bottom of the pool, is a random event, the behavior of heavy liposomes is not. It is governed by the laws of physics according to the newly acquired gravimetric properties (Chakrabarti *et al.,* 1990, 1992; Veiro and Cullis, 1990). The liposomes that entrap the solution with a specific gravity heavier than that of the surrounding media must inevitably submerge and congregate at the bottom of a pool, where they become protected from the damaging UV radiation by the water.

To survive the distraction from UV radiation, a liposome vesicle formed at the water surface must submerge during the dark hours of the nighttime to a depth where the UV intensity will be sufficiently attenuated due to absorption in water.

How deep should a liposome be submerged in water to be protected from UV? In other words, what is the water thickness sufficient to attenuate UV (≥99%)?

UV attenuation strongly depends on the water composition, particularly on the concentration and type of solutes (Ranjan *et al.,* 2022). While pure fresh water is largely transparent for UV (*e.g.,* Su and Yeh, 1996; Ranjan *et al.,* 2022), Cleaves and Miller demonstrated that UV flux is attenuated down to 1% of the initial value by just 2 mm of ocean water (Cleaves and Miller, 1998). Similar results were obtained in experiments with sedimental waters: the UV light is extinguished to 1% of the incident level by about 1 mm of sedimental waters (Garcia-Pichel and Bebout, 1996), or by water with a 2.5 g/L solution of FeCl_3_ (Gómez *et al.,* 2007). While an accurate extrapolation from the above experiments to the prebiotic conditions is difficult, let us assume that the necessary “safe” depth is about 10 mm.

How long would it take for a liposome to submerge to that depth to be protected from UV?

A terminal (settling) velocity of an object gravitationally falling in the water is determined by the balance between the gravitational force, $F_{g}$, buoyancy force, $F_{b}$, and the frictional force, $F_{d}$. For a spherical vesicle, $F_{g}=\frac{4}{3}\pi \rho_{v}gR^{3}$, $F_{b}=\frac{4}{3}\pi \rho_{w}gR^{3}$, and $F_{d}=6\pi \mu Rv$, where $\rho_{v}$ and $\rho_{w}$ are the vesicle and water mass densities, respectively, *R* and *v* are the vesicle radius and the velocity, and μ is the water dynamic viscosity coefficient. From that, the settling velocity is given by:

$$v=\frac{2}{9}\frac{\rho_{v}-\rho_{w}}{\mu }gR^{2}$$

Taking a typical water viscosity $\mu =10^{-3}kgm^{-1}s^{-1}$, obtain in practical units:

$$v\left[mm∕s\right]=2.2\times 10^{-3}\left(\rho_{v}∕\rho_{w}-1\right)R^{2}\left[\mu m\right]$$

**FIG. 3.**
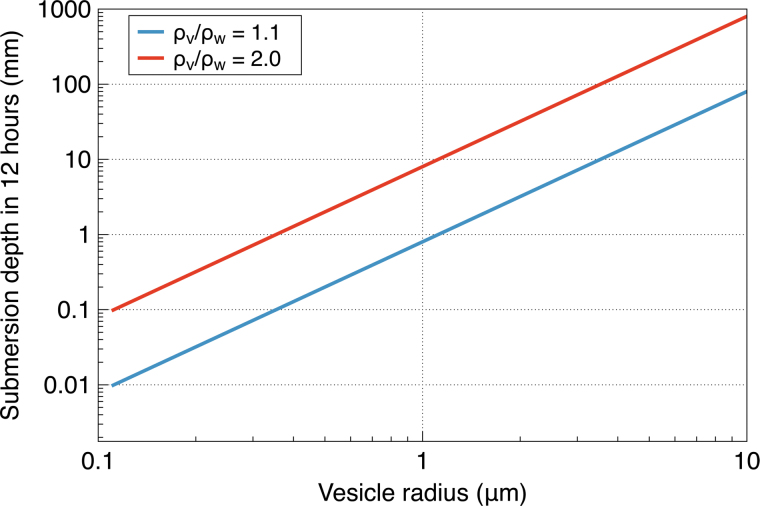
The submersion depth versus the vesicle radius for “light” vesicles with a relative specific density of $\rho_{v}∕\rho_{w}=1.1$ and “heavy” vesicles with a relative specific density of $\rho_{v}∕\rho_{w}=2.0$.

Assume that the vesicles continuously submerge for the 12 h of nighttime darkness. The submersion depth versus the vesicle radius is shown in Fig. 3 for “light” vesicles with a relative specific density of $\rho_{v}∕\rho_{w}=1.1$ and “heavy” vesicles with a relative specific density of $\rho_{v}∕\rho_{w}=2.0$.

We see that light vesicles with a radius greater than 3 μm are able to submerge at a depth of 10 mm during the nighttime, while for heavy vesicles, the minimum required radius is lower, about 1 μm. The vesicles that are too small or too light will not have enough time to submerge to that depth and will be destroyed by UV when the Sun comes up. Larger and heavier vesicles will submerge to that “safe” depth and will be protected from UV.

The important point is that the terminal velocity is proportional to the square of the radius of liposomes; thus the submerging time is inversely proportional to that. That would substantially increase the survivability of larger liposomes.

Therefore, based on these calculations and experimental observations (Garcia-Pichel and Bebout, 1996; Cleaves and Miller, 1998; Ranjan *et al.,* 2022; Gómez *et al.,* 2007), we can suggest that (1) nighttime (12 h) is sufficient for the submergence of heavy liposomes in primordial water to a depth of about 10 mm, which should offer complete protection from the damaging UV radiation, and (2) the negative buoyancy of liposomes can constitute a fitness feature under UV selective pressure that favors heavier/larger liposomes.

At this point, we would like to change the dwelling of the hypothesized events from the bottomless ocean (hydrothermal vents) to the localized fields emerging above sea level, such as hot springs, boiling pools, geysers, fumaroles, and shallow aqueous pools associated with these hydrothermal fields (Mulkidjanian, 2011; Damer and Deamer, 2015; Deamer, 2017). In this new scenario, all submerging heavy liposomes will not be scattered in the water depth; but eventually, all appear spatially in the same plane, that is, congregate at the bottom of a pool and become protected from the damaging UV radiation during daytime.

What could be the longevity of liposomes if the UV destruction force is removed from the scenario?

Experimental observation showed that liposomes could maintain their integrity in the nonfrozen aqueous media from 60 days (Hsieh *et al.,* 2002; Kim *et al.,* 2004) to a year (Sakai *et al.,* 2000; Zana, 2005; Tokuno *et al.,* 2016), even at +40°C (Sakai *et al.,* 2000). Therefore, we can hypothesize similar longevity of prebiotic liposomes, which allows the hypothesis to apply an important concept of the Lipid World model: autocatalytic replication of amphiphiles, their mutation, and selection of the fittest amphiphilic assemblies. (Note: amphiphilic assemblies' replication, mutations, and selections can occur any time after the emergence of such assemblies since these processes occur in nanoseconds' time range [Kahana and Lancet, 2021; Kahana *et al.,* 2022]; however, the evolved novelties will not survive beyond 12 h at the water-air interface.)

It is important to note that the above longevity of liposomes was observed with a single species of amphiphiles in the laboratory, which is an unlikely scenario for the prebiotic conditions. Numerous analyses suggested a heterogeneous repertoire of amphiphiles under prebiotic conditions (Chen *et al.,* 2004; Zhu and Szostak, 2009; Szostak, 2011); furthermore, the heterogeneous amphiphiles showed the most advantages in a simulation study with the GARD model (Lancet *et al.,* 2018).

A compositional design of amphiphilic micelles and the liposomal membrane was elaborated in the model of heterogeneous amphiphilic assemblies forming a mutually catalytic network (Segré *et al.,* 2000, 2001), based on the concept pioneered by Kauffman (1971, 1993). Catalytic self-replication of amphiphilic assemblies was analyzed theoretically (Farmer *et al.,* 1986; Kauffman, 1986; Segré *et al.,* 1998b) and was shown in experiments (Lee *et al.,* 1997). The model of noncovalent amphiphilic assemblies acting as transporters of compositional information and capable of undergoing replication, mutation, selection, and evolution were elucidated in numerous contributions by Lancet's group (*e.g.,* Segré *et al.,* 1998a, 1998b, 2000, 2001; Segré and Lancet, 2000; Markovitch and Lancet, 2011). Particularly, the detailed behavior of liposomes' constituents, in light of the origin of life and evolution, was analyzed by Lancet and coauthors in a simulation study using the metabolic graded autocatalysis replication domain (M-GARD) model (Lancet *et al.,* 2018).

It is logical to suggest that among the inherited variations of amphiphilic assemblies certain variations will constitute the most resilient membrane design and facilitate maximum liposomal longevity in the environment. Alternatively, other variations of amphiphilic assemblies would fail to provide lasting membrane integrity, resulting in liposomal breakdown and amphiphiles returning to the water-air interface.

Our hypothesis suggests that the heavy liposomes that acquired a resilient membrane would be aggregated at the bottom of a pool, staying in physical contact with each other for a long time. It is well-known that physical contact between liposomes resulted in liposomal fusion/growth (Nir *et al.,* 1983; Connor *et al.,* 1984; Noguchi and Takasu, 2001; Deshpande *et al.,* 2019), exchange of contents and membranes (Hanczyc and Szostak, 2004; Chan *et al.,* 2008; Yang *et al.,* 2009; Hardy *et al.,* 2015) and fission/split (Döbereiner *et al.,* 1993; Deshpande *et al.,* 2018; Penič *et al.,* 2020).

Since the above mechanistic processes assume an exchange of compositional information between liposomes, it is plausible that this physical interaction can also contribute to transmissible variations and selection of amphiphilic assemblies/liposomes by the processes described by Lancet's group in GARD models.

In theory, continuous variations in these two sets of compositional information must have the following consequences for the fate of the heavy liposomes:

(1) Evolved liposomes retain heavy content but acquire a less-resilient membrane composition due to disadvantageous mutations occurring via autocatalytic replication of amphiphilic assemblies. Such assemblies would not provide membrane stability, resulting in membrane breakdown and the disintegration of the liposomes.
(2) Due to fusion/split-generated replication of both membrane and content at the bottom of the pool, newly formed liposomes lose a heavy content and acquire the content with gravimetric properties toward positive buoyancy. Inevitably such liposomes must ascend to the surface and will be destroyed by UV during daytime.
(3) Evolved liposomes retain both the heavy content and resilient membrane composition due to advantageous mutations occurring via autocatalytic replication of amphiphilic assemblies. Preservation of both sets of compositional information would protect liposomes from both UV damage and the adversities of the surrounding media, making them amendable for further modifications and evolution of autocatalytic amphiphilic assemblies with subsequent replication, mutation, selection, and growth in the absence of any intra-liposomal genetic apparatus.

Therefore, our model extends the GARD/Lipid World scenario (Segré *et al.,* 2001; Lancet *et al.,* 2018) and explains how one of the counterparts of multistage processes—liposomes—can emerge first, survive, and propagate for a long time in abundance by themselves, thereby circumventing the obstacle regarding the spontaneous occurrence in time and space of several probable events.

While the next evolutionary step—a co-emergence of RNA precursor molecules, via the endogenous or exogenous pathways—is out of the scope of our hypothesis, we would like to sketch plausible scenarios.

In the endogenous scenario, we can appeal to autocatalytic chemical networks, a phenomenon that the founding contributions (*e.g.,* Kahana and Lancet, 2021) and our model consider among major requirements for early protocell survival and evolution. There are indications that RNA monomers can be synthesized within liposomes, via the same processes that underline autocatalytic replication, mutation, and selection of amphiphilic assemblies (Xavier *et al.,* 2020). This pathway appears to be parsimonious and does not require any further steps.

As to the exogenous pathways, we can appeal to many models and experimental observations on the spontaneous synthesis of RNA precursors under prebiotic conditions: in the atmosphere (*e.g.,* Oró and Kimball, 1961, 1962; Nguyen *et al.,* 2015; Becker *et al.,* 2016; Ferus *et al.,* 2017; Rodriguez *et al.,* 2019); at the water-air interface (*e.g.,* Nam *et al.,* 2018); and in water (*e.g.,* Cafferty *et al.,* 2016; Joyce and Szostak, 2018).

However, within the exogenous pathways a question arises: What is the probability of interaction between dispersed RNA precursors and liposomes? We believe that in our model there are two scenarios with this probability being very high.

First scenario: Considering the continuous synthesis of phospholipids in primordial water (Liu *et al.,* 2020) and Langmuir monolayer formation (Kaganer *et al.,* 1999; Giner-Casares *et al.,* 2014), the RNA precursors that are synthesized in the atmosphere, or at the water-air interface, would come in contact with Langmuir monolayer. During the nighttime liposomogenesis, these precursors would be encapsulated and, together with other heavy molecules, would serve as initial heavy cargo for the submergence of liposomes.

Second scenario: Since RNA precursors have a higher specific gravity than water, for example, a ribose (Zhao and Wang, 2021), these precursors must come in contact with the water surface and, over time, descend into the water. What would be a hypothetical depth-point where RNA precursors would be concentrated? It is the bottom of the pool, which, in our model, already housed the heavy liposomes. We can further invoke the observation of spontaneous extraction of molecules from surrounding media by liposomes (de Souza *et al.,* 2014; Sugiyama *et al.,* 2020).

Providing the retention of a heavy content and continuous autocatalytic replication, mutation, and selection of amphiphilic assemblies toward membrane stability, as elucidated in numerous contributions by Lancet's group, we can hypothesize extended longevity of the heavy liposomes and the longevity of the population of heavy liposomes hence much greater. Since all heavy liposomes are in the same plane exchanging content (Hanczyc and Szostak, 2004; Chan *et al.,* 2008; Yang *et al.,* 2009; Hardy *et al.,* 2015), an accidental assembly of RNA precursors into functional molecules became plausible, especially considering the lipid-assisted synthesis of RNA-like polymers (Rajamani *et al.,* 2008).

## The Major Points of the Hypothesis

**FIG. 4.**
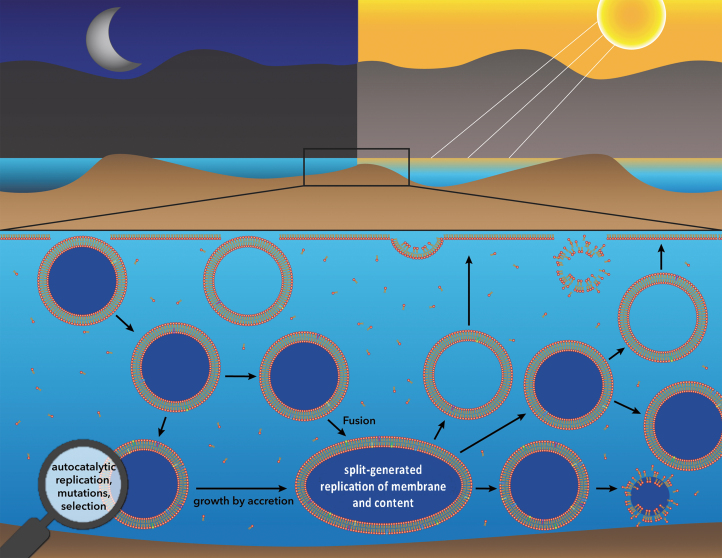
Self-sustained Darwinian evolution of liposomes driven by solar UV radiation, day/night cycle, and membrane stability. Enzyme-free synthesized phospholipids ascend to the water-air interface, forming phospholipid layers and liposomes. Liposomes formed at nighttime accidentally entrap heavy solutes (due to the presence of Miller-Urey molecules, natural primordial heavy molecules, and RNA precursors, *e.g.,* a ribose, all depicted in dark blue), descend to the bottom of the pool, and become protected from UV. The longevity of the protected heavy liposomes is sufficient for selecting the membrane design of the highest stability, which is achieved via autocatalytic replication of amphiphiles and through their mutation and selection. Heavy liposomes at the bottom of the pool undergo fusion/split-generated division and exchange of both membrane and content components. Descendant liposomes that lose heavy content ascend to the water surface and are decomposed by UV at daytime. Descendant heavy liposomes, that lose membrane resilience due to disadvantageous mutations of amphiphilic assemblies, are disintegrated regardless of UV action. Only liposomes that preserve both sets of compositional information (heavy content and resilient membrane) are protected from UV damage and adversities of surrounding media and can undergo further replication and division processes, forming a population of evolving liposomes.

(1) We extend the GARD/Lipid World model by proposing a novel scenario of self-sustained Darwinian evolution of the liposomes driven by the following ever-present natural phenomena: solar UV radiation, day/night cycle, and gravity.
(2) Natural synthesized phospholipids in water inevitably form a Langmuir monolayer at the air-water interface, which is followed by formation of a floating phospholipid bilayer and liposomes.
(3) The solar UV radiation inevitably decomposes the liposomes into separate phospholipid molecules, which return to the Langmuir monolayer. This destruction and recycling are repeated every time the Sun comes up.
(4) The only possibility for liposomes to survive the daytime UV destruction is to acquire negative buoyancy by encapsulating heavy solutes and submerge, becoming shielded from UV by the water.
(5) The longevity of the prebiotic liposomes in nonfrozen aqueous media is sufficiently long to assume the autocatalytic replication of amphiphiles, their mutation, and selection of the fittest amphiphilic assemblies, which provides a resilient membrane design and facilitates maximum liposomal longevity in the environment, as shown in GARD models by Lancet's group.
(6) Only liposomes that preserve both sets of compositional information (heavy content and resilient membrane) are protected from UV damage and surrounding media adversities. With the continuous source of phospholipids and perpetuation of the autocatalytic processes, the congregation of heavy liposomes at the bottom of the pool grows, forming the self-sustain evolving population, thereby establishing Darwinian progression in the absence of a genetic apparatus.
(7) Temporary and spatial expansion of this evolving liposomal population provides the basis for the next evolutionary step: synthesis of RNA monomers via the already extended autocatalytic networks or via an accidental entrapment of RNA precursor molecules, both resulting in the assembly of RNA precursors into complex functional molecules, such as ribozymes/RNA. The sketch of the hypothesized events is depicted in Fig. 4.

## Proposed Experiments

The hypothesis can be tested by following experiments.

(1) Measure UV absorption in water solution of FeCl_3_ crystals versus their concentration.
(2) Investigate the submergence of giant liposomes 10 μm in diameter in the abovementioned FeCl_3_ solution. In particular, understand the dependence of the submergence dynamics versus the mass density of the liposomes and the history of UV irradiation.

## Concluding Remarks

In this work, we offer a new model of the self-sustained Darwinian evolution of liposomes. The model can be viewed as an extension of the Lipid World hypothesis, to which it adds the contribution of the following ever-present natural events we believe to be essential for the origin of life:

(1) Solar UV radiation, as a negative selection force acting on the liposome.
(2) The negative buoyancy of liposomes and membrane stability as the driving forces behind the development of fitness features countering the negative selective pressure of the UV and aqueous adversities.
(3) The day/night cyclicity, that through the ever-repeatable rotation of the adverse selection forces and conditions enables the favorable evolution of the fitness features.
(4) Our model considers the water-air interface and the bottom of the pool as two spatial planes crucial for life's origin, echoing the idea of the “subvital areas” of Earth's surface expressed by Rutten and Oparin (Rutten, 1971; Oparin, 1974).

## Credit Statements

Vladimir Subbotin: Initial conceptualization, formal analysis, visualization, writing—original draft, writing—review and editing.

Gennady Fiksel: Elaboration of the hypothesis, methodology, terminal velocity model, validation, mathematical analysis, writing—original draft, writing—review and editing.

## Abbreviations Used

MUm: Miller-Urey molecules
NPHm: Natural Primordial Heavy molecules

## Notes

Associate Editor: Norman Sleep
